# To screen or not to screen for peripheral arterial disease in subjects aged 80 and over in primary health care: a cross-sectional analysis from the BELFRAIL study

**DOI:** 10.1186/1471-2296-12-39

**Published:** 2011-05-23

**Authors:** Stein Bergiers, Bert Vaes, Jan Degryse

**Affiliations:** 1 Interuniversitair Centrum voor HuisartsenOpleiding (ICHO), Kapucijnenvoer 33, bus 7001, 3000 Leuven, Belgium; 2 Institute of Health and Society, Université Catholique de Louvain, Clos Chapelle-aux-Champs 30, bte 3005, 1200 Brussels, Belgium

## Abstract

**Background:**

Peripheral arterial disease (PAD) is common in older people. An ankle-brachial index (ABI) < 0.9 can be used as an indicator of PAD. Patients with low ABI have increased mortality and a higher risk of serious cardiovascular morbidity. However, because 80% of the patients are asymptomatic, PAD remains unrecognised in a large group of patients. The aims of this study were 1) to examine the prevalence of reduced ABI in subjects aged 80 and over, 2) to determine the diagnostic accuracy of the medical history and clinical examination for reduced ABI and 3) to investigate the difference in functioning and physical activity between patients with and without reduced ABI.

**Methods:**

A cross-sectional study embedded within the BELFRAIL study. A general practitioner (GP) centre, located in Hoeilaart, Belgium, recruited 239 patients aged 80 or older. Only three criteria for exclusion were used: urgent medical need, palliative situation and known serious dementia. The GP recorded the medical history and performed a clinical examination. The clinical research assistant performed an extensive examination including Mini-Mental State Examination (MMSE), Geriatric Depression Scale (GDS-15), Activities of Daily Living (ADL), Tinetti test and the LASA Physical Activity Questionnaire (LAPAQ). ABI was measured using an automatic oscillometric appliance.

**Results:**

In 40% of patients, a reduced ABI was found. Cardiovascular risk factors were unable to identify patients with low ABI. A negative correlation was found between the number of cardiovascular morbidities and ABI. Cardiovascular morbidity had a sensitivity of 65.7% (95% CI 53.4-76.7) and a specificity of 48.6% (95% CI 38.7-58.5). Palpation of the peripheral arteries showed the highest negative predictive value (77.7% (95% CI 71.8-82.9)). The LAPAQ score was significantly lower in the group with reduced ABI.

**Conclusion:**

The prevalence of PAD is very high in patients aged 80 and over in general practice. The clinical examination, cardiovascular risk factors and the presence of cardiovascular morbidity were not able to identify patients with a low ABI. A screening strategy for PAD by determining ABI could be considered if effective interventions for those aged 80 and over with a low ABI become available through future research.

## Background

Peripheral arterial disease (PAD) refers to atherosclerotic occlusive disease of the arterial system distal to the aortic bifurcation and is a relatively common disorder in older persons [1]. A Dutch study found a PAD prevalence of 19.1% in subjects aged 55 and older, and this prevalence increased with age [1]. The clinical signs of PAD have a significant impact on quality of life, ranging from intermittent claudication to pain symptoms at rest, arterial ulceration, necrosis and even gangrene in the later stages [2]. In agreement with the approach followed by Fowkes et al [3] and by Schroll and Munck [4] PAD was considered present when the ABI was lower than 0.90 in at least one leg, a threshold value used in most studies [1,2,5,6].

PAD is often a subclinical disease in which the patient does have an ABI of less than 0.9 yet experiences no symptoms. Moreover, only 22% of patients with PAD experience symptoms [7]. PAD may also be regarded as a marker for cardiovascular disease in people over 65 years. The lower the ABI, the more likely it will be signs of atherosclerosis are present [8]. Also, patients with subclinical PAD more frequently have signs of cardiovascular disease, such as hypertension, carotid stenosis, concomitant ECG abnormalities, angina pectoris or a previous history of myocardial infarction [9]. Diehm et al. showed that patients with PAD have increased mortality and a higher risk of serious cardiovascular morbidity [10-14].

The conventional way of calculating ABI is the Doppler method, in which the quotient between the blood pressure measured at the ankle and the blood pressure measured at the arm of the patient is calculated. However, in everyday practice, this technique is not frequently used [15]. This method demands regular practice and an experienced researcher or clinician [16]. Recently, a new method has been developed for the calculation of ABI using an automatic oscillometric blood pressure reading of the arm and the ankle. This method is simple to carry out, requires less training and has been validated in several studies [17-20].

However, patient groups for whom ABI measurement would be useful have not yet been clearly defined. Furthermore, uncertainty still exists concerning the need to screen for PAD; however, because 80% of the patients are asymptomatic, a large group of patients remain unrecognised [21,22]. Moreover, subjects aged 80 and over are often underrepresented in studies or even excluded by design. There is a growing consciousness that study results from younger patient groups cannot simply be extrapolated to the oldest old [23].

The aims of this study were 1) to determine the prevalence of reduced ABI (< 0.9) in subjects aged 80 and over, 2) to discover whether a medical history and clinical examination can identify patients with reduced ABI and 3) to investigate the difference in function and physical activity between patients with and without reduced ABI.

## Methods

### BELFRAIL study

This cross-sectional study was embedded within the BELFRAIL study. The BELFRAIL study is a prospective, observational, population-based cohort study, and its aim is to gain improved insight into the epidemiology and pathophysiology of chronic diseases in subjects aged 80 and over [24]. The study protocol has been approved by the Biomedical Ethics Committee of the Medical School of the Université Catholique de Louvain of Brussels (B40320084685).

### Patient selection

**Figure 1:**
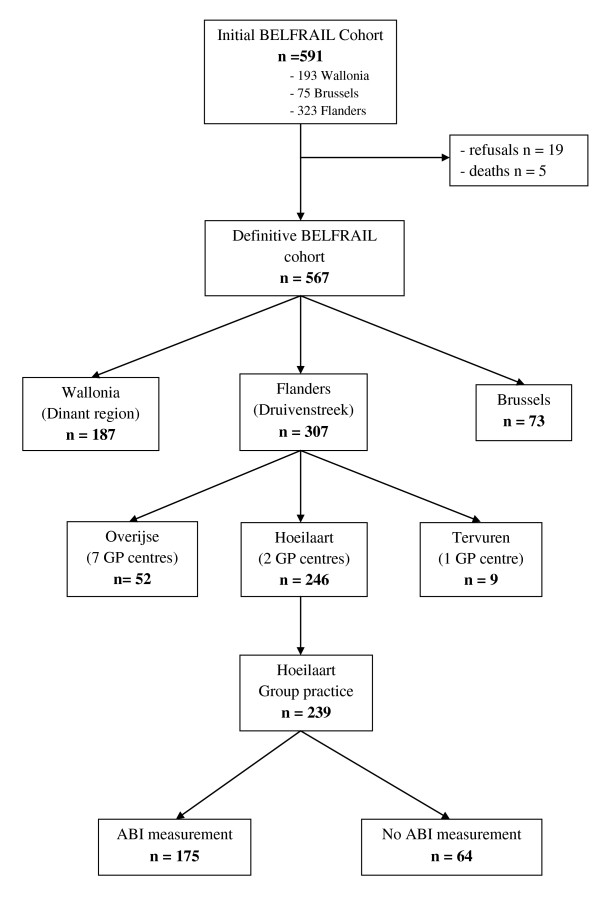
**BELFRAIL study participants flow-chart**.

After the initial cohort was constituted (n = 593), 19 subjects refused to continue and five died before the examinations were started (Figure 1). A total of 567 patients from three different regions of Belgium: the Dinant region (17 GP practices), Brussels (2 GP practices) and the Druivenstreek (10 GP practices) were included in the definitive BELFRAIL cohort. For this study only the patients from the GP practice of Hoeilaart (Druivenstreek) were selected (n = 239).

This GP practice consists of five doctors and two trainee GPs. Between March 2009 and July 2009, every patient aged 80 or older who came in for a consultation or was visited at home (including care homes and retirement homes) was invited to participate in the BELFRAIL study. Only three criteria for exclusion were used: urgent medical need, palliative situation and known serious dementia (MMSE < 15/30).

After informed consent (by the patient and the caregiver if MMSE < 18/30) the participating patients were examined by the GP and a clinical research assistant (CRA). The CRA was a nurse, a psychologist or a biomedical scientist and performed an extensive examination including performance testing and questionnaires following a standardised protocol.

### Examination by the GP

The GP recorded known cardiovascular risk factors and the previous medical history of the patient. Indicators of the cardiovascular risk profile were hypertension, hyperlipidaemia, type 2 diabetes mellitus, smoking (either current or ex-smoker) and BMI > 30. Cardiac morbidities were defined as a positive response for the history of angina pectoris or acute myocardial infarction, an episode of decompensated heart failure, chronic atrial fibrillation or other rhythm disturbances and a previous history of PTCA or stent and coronary surgery. Other cardiovascular morbidities consisted of previous TIA or CVA, known PAD and a previous history of arterial surgery. The GP then carried out a standardised clinical examination. The pulses of the lower limbs were palpated at the point of the arteria dorsalis pedis or arteria tibialis posterior. If the artery was palpable the clinical examination was negative, if not it was positive.

### Investigation by the CRA

#### Mini-Mental State Examination (MMSE)

The MMSE tests the cognitive function of the patient with a score ranging from 0 to 30 (maximum score). Orientation in time and space is tested, as are memory, calculation, understanding and constructive praxis [25].

#### Geriatric Depression Scale (GDS-15)

This questionnaire was specifically developed for older patients and consists of 15 questions that assess the functional and mood-associated symptoms of depression [26-28].

#### Activities of Daily Living (ADL)

Functional limitations are assessed by determining the degree of difficulty of carrying out six everyday activities. The answers vary between (1) "No, I cannot do this" to (5) "Yes, without any problems". The total score varies between 6 (minimum) and 30 (maximum) [29].

#### Tinetti test

This consists of a number of tests concerning movement and balance. Individual aspects are scored between 0 and 2, for a total score of 28 points. This test detects patients with an increased risk of falling [30].

#### LASA Physical Activity Questionnaire (LAPAQ)

The LAPAQ questionnaire assesses the frequency and duration of walking outside, cycling, gardening, light and heavier housework and sporting activities over the previous two weeks. The duration of each of these activities is investigated and scored. The total LAPAQ score is calculated by multiplying the frequency and duration of the various aspects by one another [31].

#### Performance tests

The number of seconds the patient requires to complete each test is recorded [32].

• *Performance test 1*: walking test: the patient is asked to walk as quickly as they can for 3 metres, then turn and walk back 3 metres.

• *Performance test 2*: chair test: the patient is asked to stand up and sit down again five times with their arms crossed as quickly as possible.

• *Performance test 3*: cardigan test: the patient is asked to remove a cardigan as quickly as possible.

### Calculating ABI

At the completion of the examination, ABI was measured by the GP with the aid of an automatic oscillometric appliance (SCVL-2007, Diegel Healthworks^® ^and associated software). Participating GP's were trained to standardise the ABI measurement. The patient was placed in the supine position, and a pressure cuff was placed around the arm and leg, first on the left side and then on the right side. The pressure cuffs were simultaneously, automatically inflated by the software, and both the systolic and the diastolic blood pressures were measured at the arm and leg. ABI was calculated as the quotient between the systolic blood pressure at the ankle and the systolic blood pressure at the arm. A value of 0.9 or more was viewed as normal, a value of less than 0.9 was considered a reduced ABI [5]. Afterwards, the ABI measurement was reported back to the treating GP and all physicians were referred to existing guidelines on PAD for follow-up [2].

### Data analysis

Differences between patients with and without a recorded ABI and between patients with and without a reduced ABI were calculated with the independent Student's *t*-test and the Mann-Whitney *U *test for non-normally distributed variables. A *P*-value of < 0.05 was considered statistically significant.

The Jonckheere-Terpstra test was used to compare the median ABI values between different categories of patients with increasing numbers of cardiovascular risk factors and cardiovascular morbidities.

The sensitivity, specificity, negative predictive value (NPV) and positive predictive value (PPV) of the medical history and the clinical examination for an ABI of < 0.9 were calculated with the help of 2 × 2 tables. At the same time, we calculated the number of reduced ABI values we would have missed and the number of negative examination results we would have gotten if we had used the medical history and clinical examination to select patients for ABI measurement.

## Results

### Patient population and prevalence

In Hoeilaart 239 patients participated in the BELFRAIL study (Figure 1). In 175 of the participants, an ABI was calculated for at least one leg. In the remaining 64 participants, ABI was not measured for a variety of reasons, including pain in the leg when the cuff was inflated, extensive oedema of the lower leg, and a failed oscillometric blood pressure reading in the lower leg despite repeated attempts.

Table 1 lists the differences between patients with and without ABI readings. The patients who did not have any readings taken were institutionalised more frequently (*P *< 0.10) and on average had a lower level of education (*P *< 0.10). MMSE score was also lower in the group who were not examined (*P *< 0.05). There was no difference in the clinical characteristics between the two patient groups.

**Table 1:** Comparison between patients with and without an ABI recorded.

	ABI measured		P*
		
	**Yes (*n *= 175**)	No (*n *= 64)	
Sociodemographic characteristics			
Men (*n*, %)	64 (36.6)	20 (31.2)	0.45
Age (mean ± SD)	84.9 ± 3.9	85.1 ± 3.7	0.78
Institutionalised (*n*, %)	10 (5.7)	9 (14.1)	0.081
≤ Lower secondary education (*n*, %)	130 (74.3)	54 (84.4)	0.076
Functioning			
MMSE (median, IQR)	28 (26 - 29)	27 (24 - 29)	0.011^£^
ADL (median, IQR)	25 (21 - 27)	25 (20 - 27)	0.96^£^
GDS-15 (median, IQR)	2 (1 - 4)	2 (1 - 3)	0.32^£^
Performance tests (median, IQR)	8 (5 - 11)	9 (4 - 12)	0.57^£^
LAPAQ (median, IQR)	84.0 (38.5 - 106)	71.5 (44.5 - 106.5)	0.64^£^
Tinetti (median, IQR)	27 (25 - 28)	27 (25 - 28)	0.82^£^
Clinical characteristics			
Hypertension (*n*, %)	131 (74.9)	47 (73.4)	0.83
Hyperlipidaemia (*n*, %)	70 (40.0)	28 (43.8)	0.60
Diabetes (*n*, %)	36 (20.6)	18 (28.1)	0.24
Smoker, current or ex- (*n*, %)	55 (31.4)	18 (28.1)	0.63
BMI ≥30 (*n*, %)	48 (27.7)	15 (23.4)	0.51
Angina pectoris (*n*, %)	31 (17.7)	11 (17.2)	0.93
Myocardial infarction (*n*, %)	20 (11.4)	9 (14.1)	0.58
TIA or CVA (*n*, %)	32 (18.3)	10 (15.6)	0.63
Known PAD (*n*, %)	17 (9.7)	5 (7.8)	0.65
Arterial surgery (*n*, %)	8 (4.6)	2 (3.1)	0.62

* Independent Student's *t*-test; IQR, inter-quartile range; ^£^, Mann-Whitney *U *test; ^$^. ABI: ankle-brachial index; SD: standard deviation; MMSE: Mini-Mental State Examination; ADL: Activities of Daily Living; GDS: Geriatric Depression Scale; LAPAQ: LASA Physical Activity Questionnaire; BMI: body mass index; TIA: transient ischaemic attack; CVA: cerebro-vascular accident; PAD: peripheral arterial disease.

In 23 patients, ABI was measured in only one leg. ABI was measured in a total of 327 legs. For six of the legs, ABI was > 1.5, and these results were excluded from further analysis. In 94 of the 327 legs, ABI of < 0.9 (29.7%) was found. Of the 175 patients, 70 had at least one leg with an ABI of < 0.9 (40%).

### Diagnostic value of cardiovascular morbidity and a clinical examination for low ABI

**Figure 2:**
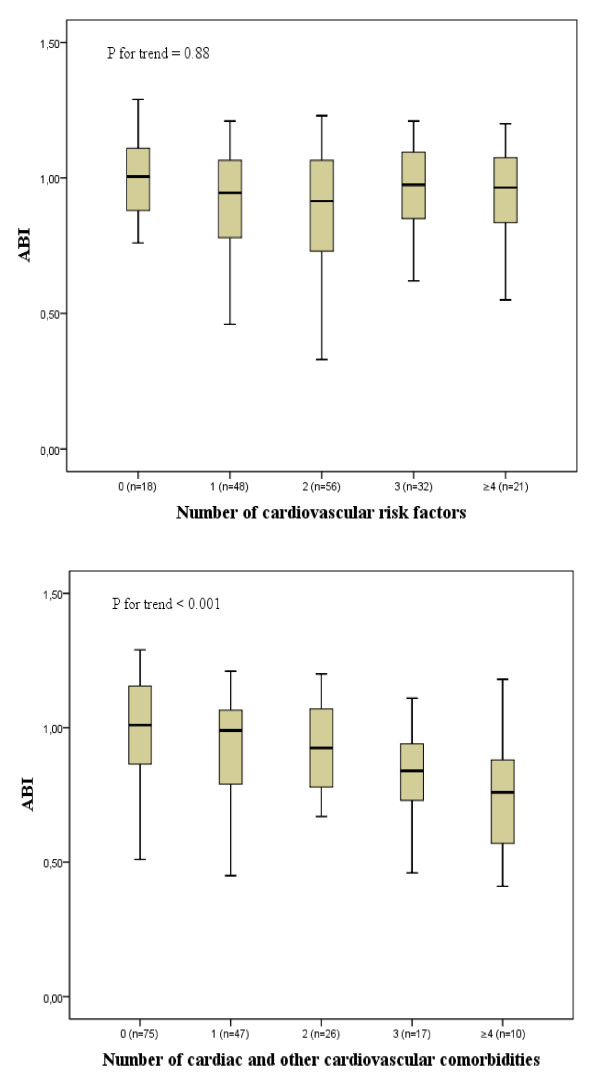
**Correlations between ABI and the number of cardiovascular risk factors and the number of cardiac and other cardiovascular comorbidities**. P for trend = Jonckheere-Terpstra test. ABI: ankle-brachial index.

Table 2 shows the differences in cardiovascular burden between patients with a low and a normal ABI. The average age was higher in the group with a low ABI. The presence of cardiovascular risk factors was prevalent but did not differ between the two groups, nor was there a correlation between the number of risk factors and ABI (Figure 2). The presence of a cardiovascular risk factor as an identifier of low ABI had a high sensitivity (92.9%) and a low specificity (12.4%) (Table 3). If ABI had been determined only for patients with at least one cardiovascular risk factor 7% (5/70) of the patients with ABI < 0.9 would have been missed and 59% (92/157) would have had a normal result.

**Table 2:** Comparison of the cardiovascular burden between patients with ABI < 0.9 and ABI ≥0.9.

	ABI < 0.9 (*n *= 70)	ABI ≥ 0.9 (*n *= 105)	P*
Men (*n*, %)	29 (41.4)	35 (33.3)	0.28
Age (mean ± SD)	85.7 ± 4.8	84.4 ± 3.2	0.044
Cardiovascular risk profile (*n*, %)	65 (92.9)	91 (88.3)	0.31
Hypertension (*n*, %)	54 (77.1)	77 (73.3)	0.57
Hyperlipidaemia (*n*, %)	24 (34.3)	46 (43.8)	0.21
Diabetes (*n*, %)	14 (20.0)	22 (21.0)	0.88
Smoker, current or ex- (*n*, %)	26 (37.1)	29 (27.6)	0.19
BMI ≥ 30 (*n*, %)	15 (21.4)	33 (32.0)	0.12
Cardiac morbidity (*n*, %)	42 (60.0)	41 (39.0)	0.006
Angina pectoris (*n*, %)	16 (22.9)	15 (14.3)	0.16
Myocardial infarction (*n*, %)	13 (18.6)	7 (6.7)	0.026
Episode of decompensated heart failure (n, %)	6 (8.6)	6 (5.7)	0.47
Chronic atrial fibrillation (*n*, %)	11 (15.7)	10 (9.5)	0.24
Rhythm disorder (*n*, %)	16 (22.9)	16 (15.2)	0.22
PTCA - stent (*n*, %)	9 (12.9)	5 (4.8)	0.077
Coronary surgery (*n*, %)	3 (4.3)	4 (3.8)	0.88
Other cardiovascular morbidity (*n*, %)	21 (30.0)	21 (20.0)	0.14
TIA of CVA (*n*, %)	12 (17.1)	20 (19.0)	0.75
PAD (*n*, %)	13 (18.6)	4 (3.8)	0.004
Arterial surgery (*n*, %)	7 (10.0)	1 (1.0)	0.018

* Independent Student's *t*-test.

ABI: ankle-brachial index; SD: standard deviation; BMI: body mass index; PTCA: percutaneous transluminal coronary angioplasty; TIA: transient ischaemic attack; CVA: cerebro-vascular accident; PAD: peripheral arterial disease.

**Table 3:** Diagnostic value of the cardiovascular risk profile, known cardiovascular history and the clinical examination for low ABI.

Test	Sens, % (95% CI)	Spec, % (95% CI)	NPV, % (95% CI)	PPV, % (95% CI)	Missed cases (%)	Negative ABI (%)
CV risk factor*, *n *≥ 1	92.9 (84.1-97.6)	12.4 (6.8-20.2)	72.2 (46.5-90.2)	41.4 (33.6-49.5)	5/70 (7.1)	92/157 (58.6)
CV morbidity*, *n *≥ 1	65.7 (53.4-76.7)	48.6 (38.7-58.5)	68.0 (56.2-78.3)	46.0 (36.0-56.3)	24/70 (34.3)	54/100 (54.0)
CV morbidity*, *n *≥ 3	27.1 (17.2-39.1)	92.4 (85.5-96.6)	65.5 (57.3-73.2)	70.4 (49.8-86.2)	51/70 (72.9)	8/27 (29.6)
Cardiac morbidity, *n *≥ 1	60.0 (47.6-71.5)	61.0 (50.9-70.3)	69.6 (59.1-78.7)	50.6 (39.4-61.8)	28/70 (40.0)	41/83 (49.4)
Other CV morbidity, *n *≥ 1	30 (19.6-42.1)	80 (71.1-87.2)	63.2 (54.4-71.4)	50.0 (34.2-65.8)	49/70 (70.0)	21/42 (50.0)
Clinical examination^$^	45.7 (35.4-56.3)	79.8 (74.0-84.9)	77.7 (71.8-82.9)	48.9 (38.1-59.8)	51/94 (54.3)	45/88 (51.1)
Cardiac morbidity* (n ≥ 1) AND Clinical examination^$^	35.1 (25.5-45.6)	87.0 (81.9-91.1)	76.1 (70.4-81.2)	53.2 (40.1-66.0)	61/94 (64.9)	29/62 (46.8)

*Prevalence of ABI < 0.90 was 40%, ^$ ^Prevalence of ABI < 0.9 was 29.7%. ABI: ankle-brachial index; Sens: sensitivity; CI: confidence interval; Spec: specificity; NPV: negative predictive value; PPV: positive predictive value; CV: cardiovascular.

The prevalence of cardiac morbidity was significantly higher in patients with low ABI. More specifically, the numbers of myocardial infarctions and cardiac stents were higher in patients with ABI < 0.9. In 13 (18.6%) patients with a low ABI, there had already been a diagnosis of PAD, and 4 (3.8%) patients with a diagnosis of PAD had a normal ABI. A negative correlation was found between the number of cardiovascular (cardiac and other) morbidities and ABI. The median ABI dropped from 1.0 [IQR 0.86-1.2] in patients with no morbidities to 0.99 [IQR 0.78-1.1] in patients with one morbidity, 0.93 [IQR 0.78-1.1] with two morbidities, 0.84 [IQR 0.68-0.95] with three morbidities and 0.76 [IQR 0.54-0.89] in patients with four or more morbidities (Jonckheere-Terpstra, *P *< 0.001) (Figure 2).

The presence of a cardiovascular morbidity had a sensitivity of 65.7% and a specificity of 48.6%. If the cardiovascular history had been used to select patients for an ABI reading, 34.3% (24/70) would have been missed and 54% (54/100) of the patients investigated would have been diagnosed with a normal ABI. If an ABI determination had been completed for only patients with three or more morbidities, the specificity and PPV of the test would have increased; however, 70% of the subjects with ABI < 0.9 would be missed.

The highest NPV was found for the clinical examination (77.7%). In 46 (26.6%) patients, no peripheral pulse was observed in the right leg; in 52 (30.1%) patients, no peripheral pulse was observed in the left leg. If the presence of peripheral pulses had been used to select patients for an ABI reading, 54% (51/94) would have been missed and 51% (45/88) would have been diagnosed with a normal ABI. Combining the cardiovascular morbidity with a positive clinical examination did not increase the NPV and only slightly increased the PPV.

### Function and physical activity

Table 4 displays the differences in function and physical activity between patients with a low ABI and normal ABI. No significant difference in function was found between the two groups. However, a trend of lower scores for everyday activities was observed in patients with ABI < 0.9.

**Table 4:** Difference in function and physical activity between patients with a low ABI and patients with a normal ABI

	ABI < 0.9 (***n *= 70**)	ABI ≥ 0.9 (***n *= 105**)	P*
Institutionalised (*n*, %)	6 (8.6)	4 (3.8)	0.19^$^
MMSE (median, IQR)	28 (26-29)	28 (25-29)	0.29
ADL (median, IQR)	25 (22-27)	24 (19-27)	0.073
GDS-15 (median, IQR)	2 (1-4)	2 (1-4)	0.41
Tinetti (median, IQR)	27 (26-28)	27 (24-28)	0.29
Performance tests			
Performance test 1 (median, IQR)	13.4 (9.4-18.9)	13.0 (9.9-16.3)	0.41
Performance test 2 (median, IQR)	14.5 (10.9-19.0)	14.2 (11.1-17.9)	0.90
Performance test 3 (median, IQR)	14.4 (11.2-17.7)	13.6 (10.5-17.8)	0.80
LAPAQ (median, IQR)	70 (28-99)	88 (48-108)	0.023

*Mann-Whitney *U *test; ^$^, independent Student's *t*-test.

ABI: ankle-brachial index; MMSE: Mini-Mental State Examination; IQR: inter-quartile range; ADL: Activities of Daily Living; GDS: Geriatric Depression Scale; LAPAQ: LASA Physical Activity Questionnaire.

The performance tests, which were short exertion tests, did not reveal differences between the patients with a normal and low ABI.

The LAPAQ score, which recorded more extensive exertions, did show a significantly lower score in the group with a low ABI.

## Discussion

### Patient population and prevalence

This study was carried out on a representative sample of people aged 80 and older as more than 95% of the population in Belgium is reported to consult the same GP or practice in case of health problems and more than 90% of people aged 65 and older have at least one contact with their GP every year, with an average of 11.9 contacts per year at the age of 75 and older [33]. In 40% of the patients investigated, a low ABI was found in at least one leg. This prevalence is somewhat greater than the prevalence found in other studies. Studies involving exclusively subjects aged 80 and over are limited. A Finnish study found a prevalence of 22% in those aged 90 and over [34]. Coni et al. found a prevalence of 19.7% in the 80-84 age group and 40% in those aged 85 and over [35]. All of these were small studies on a limited number of oldest old patients. In a larger cohort study Diehm et al. confirmed PAD in 141 of 545 patients between the ages of 80 and 84 (25.9%) and in 42 of 130 of patients over 85 (32.3%) [10].

### Diagnostic value of cardiovascular comorbidity and clinical examination for low ABI

This study found no correlation between a low ABI and the presence of cardiovascular risk factors in patients aged 80 and over. However, hypothetical selection on the basis of cardiovascular risk factors did provide an acceptable NPV and a low number of missed cases. This is predominantly explained by the high prevalence of risk factors in this age group; thus, this selection method results in a large number of negative examinations. These findings agree with the results from the Leiden 85-Plus study, which demonstrated that the Framingham risk factors no longer succeed in predicting cardiovascular mortality for patients over the age of 85 [36]. These results might indicate that classic cardiovascular risk factors can no longer be used for predicting cardiovascular morbidity and mortality in the elderly.

However, a difference was found in the prevalence of cardiovascular morbidity between the two ABI groups. As the number of cardiovascular morbidities increased, the likelihood of measuring a low ABI increased. In contrast, it was difficult to identify patients with ABI < 0.9 on the basis of known cardiovascular morbidity. Furthermore, an increase in the number of cardiovascular morbidities did not increase the diagnostic value.

The clinical examination of the GP showed an acceptable specificity and NPV; however, if the absence of peripheral pulses had been used to rule out a low ABI then more than 50% of the cases would have been missed. These results are in agreement with previous studies. Cacoub et al. showed that in the absence of a palpable peripheral pulse in the foot no clear conclusions could be drawn [37,38]. Khan et al. concluded in a meta-analysis that the clinical examination in itself was not sufficient to confirm or exclude PAD with any degree of certainty [39].

A low ABI in elderly people can be considered a marker for cardiovascular disease and a predictor for mortality and cardiovascular morbidity [8-14]. As the presence of known cardiovascular morbidity did not provide the ability to differentiate between patients with a low and a normal ABI, a low ABI could possibly be an indicator of unrecognised cardiovascular disease. In this study, 24 (34.3%) patients who had a low ABI had no known cardiovascular morbidity.

The effectiveness of possible interventions after finding a low ABI in patients aged 80 and over remains unclear. The treatment of PAD consists of an optimal treatment for classic cardiovascular risk factors, such as hypertension, hyperlipidaemia, diabetes mellitus, and stopping smoking, including the use of antiaggregants and walking exercises. These measures have a proven beneficial effect on younger patients up to the age of 75 [40]; however, no intervention studies currently exist for patients aged 80 and over. On the other hand, advances in endovascular interventions have expanded the options available for the invasive treatment of PAD the last few years. Although no causal link has yet been established, major lower extremity amputation rates have fallen by more than 25% during this time period [41].

The U.S. Preventive Services Task Force (USPSTF) recommends against routine screening for PAD among asymptomatic adults in the general population because the prevalence of PAD in this group is low and because there is little evidence that treatment of PAD at this asymptomatic stage of disease, beyond treatment based on standard cardiovascular risk assessment, improves health outcomes [42]. The present study, however, showed a high prevalence of PAD, symptomatic or asymptomatic, in a selected population of subjects aged 80 and over. The identification of the presence or absence of a low ABI on the basis of known cardiovascular morbidity and a clinical examination appeared to be difficult. Moreover, it appeared that 80% of patients with a low ABI were not yet diagnosed with PAD, probably due to the great number of patients at a sub-clinical stage. Furthermore, an oscillometric ABI measurement is simple to carry out, has been validated in several studies [17-20], and could easily be integrated in a GP consultation. Therefore, a screening program for PAD among people aged 80 and over could be considered, if effective interventions resulted from further research.

### Function and physical activity

Lower-extremity function is an important predictor of future disability, mobility loss, and nursing home placement [32,43]. This study showed physical activity assessed with performance tests, which measured short periods of exertion, was the same for patients with a low and a normal ABI. This result was to be expected, as PAD normally exhibits an onset of symptoms after a longer period of exertion. The physical activity, assessed by the LAPAQ questionnaire, which records extended exertion, was significantly lower in those patients with a low ABI. This is in line with the results from the WALCS-study, in which patients with and without PAD were subjected to a 6 minute walking test. That study showed a strong correlation amongst ABI, the function of the lower limbs and the reduced mobility of the patient. Calculation of ABI detected more people with reduced mobility than the clinical criteria for intermittent claudication [44]. Therefore, a low ABI may identify persons whose high risk for mobility loss and nursing home placement might otherwise go unrecognized. These findings suggest interventions to prevent mobility loss and nursing home placement might be appropriate for persons with low ABI [44].

### Strengths and limitations

The strength of this study is that it was carried out on an unselected first-line population and is a clear reflection of everyday practice. A large number of patients, representative of people aged 80 and over in Belgium, were examined. Furthermore, this study is the first to investigate whether a GP could identify those patients aged 80 and over with a low ABI using the history of known cardiovascular disease and a clinical examination.

This study has a number of limitations. 1) The ABI measurement and the clinical examination were carried out by the same doctor. Thus, the results of the clinical examination and the ABI were not blinded. 2) No details regarding the symptoms of intermittent claudication were available. However, in 80% of the patients with a low ABI there was no previous history of PAD. 3) A low ABI is used as the 'gold standard' for PAD. Although arteriography is generally seen as the gold standard for PAD, it is apparent from the literature that a low ABI correlates well with arteriography findings; a sensitivity of 91% and a specificity of 86% were documented [6]. Moreover, the oscillometric method is a validated method for determining ABI [17-20].

## Conclusion

This study shows that the prevalence of low ABI, clinical or subclinical, is high in patients aged 80 and over in general practice.

It was clearly demonstrated that the clinical examination, the presence of cardiovascular risk factors and the presence of cardiovascular morbidity do not identify patients with a low ABI in general practice. A screening strategy for PAD using the determination of ABI could be considered if effective interventions resulted from further research for those aged 80 and over with a low ABI.

## List of abbreviations used

ABI: ankle-brachial index; PAD: peripheral arterial disease; BMI: body mass index; PTCA: percutanous transluminal coronary angioplasty; TIA: transient ischaemic attack; CVA: cerebral vascular attack; MMSE: Mini Mental State Examination; GDS-15: Geriatric Depression Scale 15; ADL: Activities of Daily Life; LAPAQ: LASA Physical Activity Questionnaire; GP: general practitioner;

## Competing interests

The authors declare that they have no competing interests.

## Authors' contributions

SB and BV drafted the manuscript. JD and BV initiated the BELFRAIL study and are responsible for its design, conduct and analysis. All authors participated in the critical revision of the manuscript. All authors read and approved the final manuscript.

## Pre-publication history

The pre-publication history for this paper can be accessed here:

http://www.biomedcentral.com/1471-2296/12/39/prepub
